# Microbubble Resonators for All-Optical Photoacoustics of Flowing Contrast Agents

**DOI:** 10.3390/s20061696

**Published:** 2020-03-18

**Authors:** Gabriele Frigenti, Lucia Cavigli, Alberto Fernández-Bienes, Fulvio Ratto, Sonia Centi, Tupak García-Fernández, Gualtiero Nunzi Conti, Silvia Soria

**Affiliations:** 1 Centro Fermi—Museo Storico della Fisica e Centro Studi e Ricerche “Enrico Fermi”, Compendio del Viminale, Piazza del Viminale 1, 00184 Rome, Italy; g.frigenti@ifac.cnr.it (G.F.); g.nunziconti@ifac.cnr.it (G.N.C.); 2 CNR-IFAC, Istituto di Fisica Applicata “Nello Carrara”, Consiglio Nazionale delle Ricerche, via Madonna del Piano 10, I50019 Sesto Fiorentino (FI), Italy; l.cavigli@ifac.cnr.it (L.C.); f.ratto@ifac.cnr.it (F.R.); s.centi@ifac.cnr.it (S.C.); 3 Laboratorio Europeo di Spettroscopia Nonlineare (LENS)—Università degli Studi di Firenze, via Nello Carrara 1, I50019 Sesto Fiorentino (FI), Italy; 4 Facultad de Ingeniería, Universidad Nacional Autónoma de México (UNAM), Ciudad de México C.P. 04510, Mexico; afernandezbienes@gmail.com; 5 Universidad Autónoma de la Ciudad de México (UACM), Prolongación San Isidro 151, Col. San Lorenzo Tezonco, México D.F. C.P. 09790, Mexico; tupak.garcia@uacm.edu.mx

**Keywords:** photoacoustics, whispering gallery mode resonators, microbubble resonators, microfluidics, characterization of nanoparticles, flow cytometry

## Abstract

In this paper, we implement a Whispering Gallery mode microbubble resonator (MBR) as an optical transducer to detect the photoacoustic (PA) signal generated by plasmonic nanoparticles. We simulate a flow cytometry experiment by letting the nanoparticles run through the MBR during measurements and we estimate PA intensity by a Fourier analysis of the read-out signal. This method exploits the peaks associated with the MBR mechanical eigenmodes, allowing the PA response of the nanoparticles to be decoupled from the noise associated with the particle flow whilst also increasing the signal-to-noise ratio. The photostability curve of a known contrast agent is correctly reconstructed, validating the proposed analysis and proving quantitative PA detection. The experiment was run to demonstrate the feasible implementation of the MBR system in a flow cytometry application (e.g., the detection of venous thrombi or circulating tumor cells), particularly regarding wearable appliances. Indeed, these devices could also benefit from other MBR features, such as the extreme compactness, the direct implementation in a microfluidic circuit, and the absence of impedance-matching material.

## 1. Introduction

In recent years, photoacoustics applications have been implemented in biology and medicine for various purposes, such as the imaging of tissues [1,2,3,4,5,6] and the combination of diagnosis and treatment (theranostics) of such tissues for specific diseases [7,8,9]. The success of photoacoustics in these fields relies on the intrinsic properties of this physical process, which combines the specificity of optical absorption with the unhindered propagation of acoustic waves in biological tissues. In the typical photoacustic (PA) configuration, an optical absorber (e.g., plasmonic nanoparticles, blood) immersed in a host material (e.g., liquids, biological tissue) is excited through a resonant optical pulse and de-excites through an articulated chain of thermoelastic processes, ultimately leading to the emission of an acoustic shockwave [10,11,12].

Typically, a piezoelectric microphone is used to detect the acoustic wave, allowing the pressure perturbation to be converted into an electrical signal which may be recorded or elaborated by a suitable electronic chain. Depending on the host material, the microphone can be immersed in it (e.g., for liquids) or placed in contact with it through an acoustic-matching medium (e.g., for biological tissue one can use echografic gel). Piezoelectric transducers are produced with precise design goals in terms of bandwidth, acceptance angle, and sensitivity, depending on their PA application. For example, in the field of PA imaging, the ideal transducer has a flat and broad frequency response in order to determine the size of the PA emitter from the acoustic frequency of its PA wave. Piezoelectric transducers are the most common PA sensors, but they leave little room for miniaturization, since this procedure causes major performance degradation [13].

In order to achieve PA detection with compact and high-performance devices, optical transducers have been investigated and have proved to be very effective [13,14,15,16]. Various optical features and various optical configurations can be exploited to achieve PA detection; however, for the scope of this article, we will just focus on the working principle of resonator-based (or interferometric) PA transducers. In general, an optical resonator (or optical interferometer) has a spectrum made of sharp resonance peaks defined by the refractive index and the size of its constitutive elements; this spectrum is typically read-out through a photodiode while exciting the resonator with a CW laser and finely moving its emission wavelength [17,18]. When a PA wave impinges on this kind of system, the refractive index or the size of the elements is perturbed, and the positions of the resonances change: these shifts allow the presence of the PA wave to be detected and are also a measurement of the PA wave intensity [13].

In this context, we recently introduced a Whispering Gallery mode (WGM) microbubble resonator (MBR) as a microfluidic platform for material inspection via their PA response, achieving all-optical PA detection and high sensitivity towards the material spectral absorbance [19]. Microbubble resonators are manufactured in house by heating a pressurized glass capillary [20,21,22] and thus producing a spherical bulge extending from the capillary stem: this bulge is the resonator itself. Since MBRs are produced from a capillary, they can be filled easily through a microfluidic circuit and have been widely used as optical sensors due to their high sensitivity towards refractive index perturbations [23,24,25,26] and mechanical perturbations. The latter property, which comes from the high mechanical quality factor of the MBR structure [27,28,29,30], is promising for PA detection since it lowers the limit of detection through constructive mechanical interference.

Here we challenge the MBR platform in a configuration compatible with a flow-cytometry setup, by letting the PA contrast agent flow through the microbubble during measurements. Flow-cytometry based on absorbance phenomena, as in photoacoustics, has recently attracted interest due to its high sensitivity, high speed, and the possibility of in vivo applications [6,31], as well as the interesting results in terms of miniaturization and label-free detection of droplets and cells in microfluidic networks which have been recently reported [32,33]. In our case, the MBR acts as both the vial containing the contrast agent and the transducer, leading to an extremely compact system without the need for impedance-matching media. As a contrast agent, we investigated gold nanorods (GNR), which are ideal for validation purposes due to their history in PA applications [34,35,36,37,38]. The pressure produced by the liquid flow inside the MBR led us to devise an analytical technique intended to decouple the environmental noise and improve the signal-to-noise ratio by tracking the frequencies associated with the MBR mechanical modes. These results were reached by exploiting the discrete mechanical spectrum of the MBR, which is a unique feature of this system and sets it apart from other optical transducers which work using the flat-frequency modulation of the refractive index.

## 2. Experiment Description

**Figure 1:**
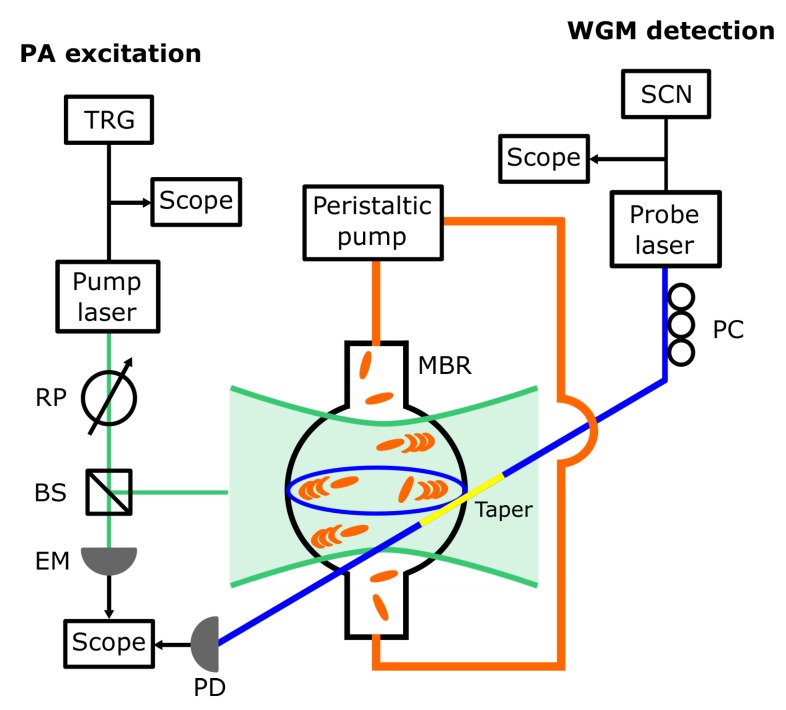
Sketch of the experimental setup. The following abbreviations are used: TRG, waveform generator used to trigger the pump laser and the oscilloscope acquisition; RP, rotatable polarizer used to set the excitation fluence; BS, beam splitter; EM, pyroelectric energy meter; SCN, waveform generator used to scan or finely set the probe laser wavelength; PC, fiber polarization controller; PD, photodiode. The microbubble resonator (MBR) area is exaggerated to highlight the MBR illumination through the pump laser and the use of a tapered fiber (taper, yellow segment of the optical fiber) to couple the probe laser to the Whispering Gallery mode (WGM).

A sketch of the experimental setup is shown in Figure 1 [19].

A home-made MBR (diameter 540 µm, volume 82 nl) was connected to a microfluidic circuit where a colloidal suspension of GNR in water was put into motion through a peristaltic pump (Minipuls 3, Gilson, Middleton, WI, USA). The GNR were synthesized through the seed-mediated approach [39,40], PEGylated and concentrated to 1 mM Au; they had an average size of 70 nm × 10 nm (length × diameter) and showed a plasmonic resonance at 1064 nm.

A pulsed free-space Nd:YAG laser (pump laser, Asclepion Laser Technologies, Jena, Germany; pulse duration 3.3 ns, repetition rate 10 Hz, pulse energy 40 µJ) emitting at 1064 nm, in resonance with the chosen set of GNR, was focused on the MBR with a spot size of 200 µm and triggered the PA emission from the GNR. The excitation fluence was set through a rotatable polarizer, while a beam splitter and an energy meter (QE8SP, Gentec-EO, Quebec, QC, Canada) were used to monitor the laser stability.

The MBR was also coupled to a home-made tapered optical fiber which allowed a WGM to be excited through a low-noise single-frequency CW fiber laser (probe laser, Koheras ADJUSTICK, NKT Photonics, Birkerød, Denmark; spectral range 1550–1551 nm). The WGM signal was detected through a gain-selectable InGaAs photodiode (PDA400, Thorlabs Newton, NJ, USA; bandwidth 10 MHz). A polarization controller was used to optimize the contrast of the WGM signal. The taper was positioned with respect to the MBR by moving it through a set of micrometer stages and observing its position with a long-working distance microscope (custom model, Navitar, Rochester, NY, USA, not shown in Figure 1).

Two waveform generators were used to trigger the emission of the pump laser and scan the wavelength of the probe laser (Keysight 33210A and Keysight 33220A, respectively, both from Agilent Technologies, Santa Clara, CA, USA). The waveform from the first generator was also used to trigger the acquisition of the photodiode and the energy meter signals via a digital oscilloscope (RTO1004, Rohde and Schwarz, Munich, Germany).

**Figure 2:**
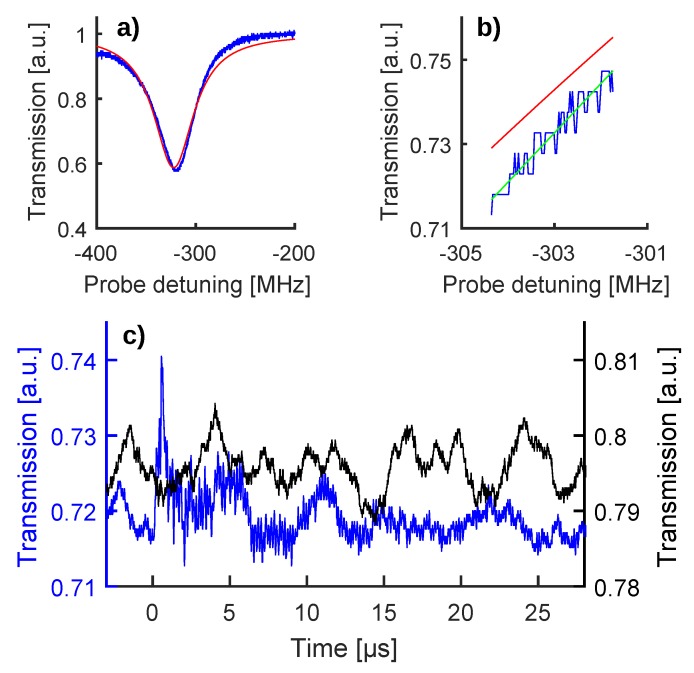
(**a**) WGM resonance used for this experiment (blue curve) along with an overall fit (red curve, theoretical shape according to [41]). (**b**) Enlargement of the half-maximum work-point, showing that the parabolic fit (green curve) allows a more faithful representation of the WGM fringe (blue curve) with respect to the overall fit (red curve). (**c**) The black curve is a baseline trace (or zero-signal trace) recorded after setting the probe laser on the half-maximum work-point and while keeping the pump laser disabled. The blue curve is a raw read-out of the photoacustic (PA) wave generated by shining the gold nanorods (GNR) with a single pump pulse, producing a 10 mJ/cm${}^{2}$ fluence. This panel uses two ordinate axes to keep the aforementioned color code.

The experiment was performed as follows:

We filled the MBR by turning the peristaltic pump on, and while keeping it on for the entire experiment, we searched a WGM resonance which showed good coupling through a wavelength scan of the probe laser. The coupling of this resonance was optimized using the polarization controller, obtaining the shape shown in Figure 2a. At variance with [19], where the peristaltic pump was off during the experiment, the resonance featured a wobble on a time scale of the order of seconds: we ascribe this wobble to the pulsating liquid flow inside of the MBR and to the tension applied on the capillary stem by the peristaltic pump.

The probe laser was then set on the half-maximum wavelength work-point and the wavelength scan stopped, obtaining a baseline trace (black curve in Figure 2c). As a result of the resonance wobble, the work-point did not remain fixed on the half-maximum and moved along the resonance fringe: this baseline movement did not constitute a problem for the experiment, since the PA signal evolved on a much shorter time scale, as is shown in the following. It is important to highlight that these baseline traces represent the zero signal of the experiment, since they were recorded while the pump laser was disabled and therefore no PA wave was generated.

Finally, the pump laser was enabled and the WGM read-out changed to a shape shown as a blue curve in Figure 2c, featuring a sharp peak followed by fast oscillations. These oscillations are caused by the shift of the WGM resonance frequency induced by the PA wave triggered through the pump laser [19]. This interpretation is corroborated by the clear PA signal shown in Figure S1 of the Supplementary Materials, which was obtained after immersing the MBR in water, filling it with GNR, coupling it with a standard US transducer (Olympus Panametrics, mod V382-SU-F, sensor diameter 0.5 inch, frequency range 3.5 MHz, focal distance 0.83 inch, 40-dB amplifier mod 5676, Tokyo, Japan), and triggering PA emission with the same pump laser [40,42].

## 3. Data Analysis and Results

**Figure 3:**
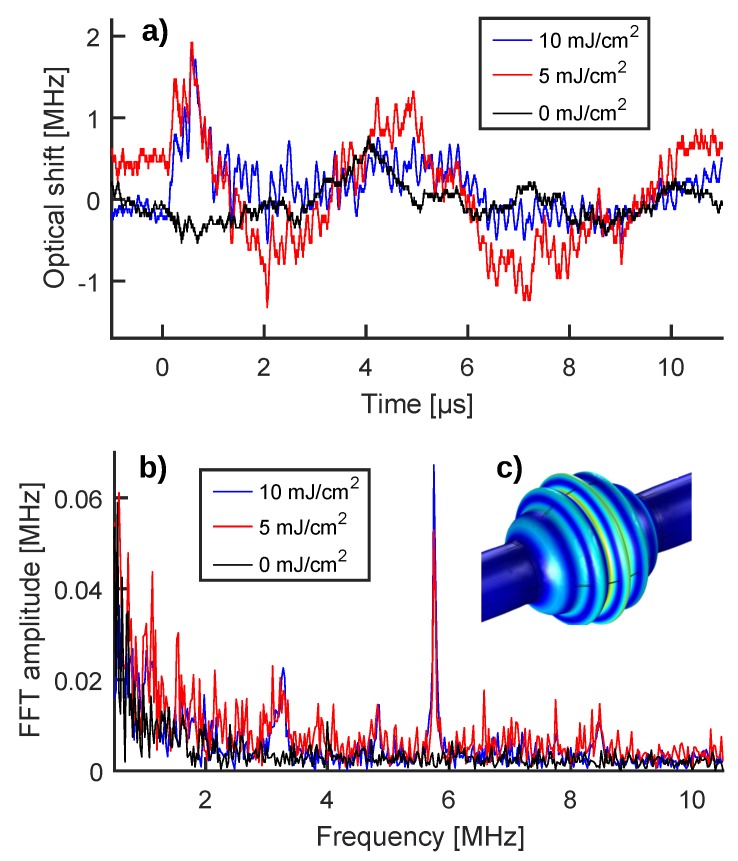
(**a**) Optical shifts induced in the WGM resonance by environmental noise (black curve) and by a PA wave (blue curve) as deduced from the raw transmission signals shown in Figure 2c keeping the same color code. The shift produced by a 5 mJ/cm${}^{2}$ fluence pulse is added for comparison (red curve). (**b**) Fourier spectrum of the optical shifts reported in panel (a), keeping the same color code. (**c**) Deformations of the MBR walls (in arbitrary units) for a breathing mode close to 5.75 MHz, as deduced by solving the eigenvalues problem for the MBR system through COMSOL Multiphysics^®^. Blue means less deformation, while yellow means more deformation.

To deduce the optical shift induced in the WGM resonance by the PA wave, the transmission oscillations of the read-out traces had to be converted into detuning oscillations. This conversion was based on the WGM shape recorded before enabling the pump laser (Figure 2a) and consisted in approximating the WGM fringe to a parabola around the half-maximum work-point (Figure 2b). This empirical method (green trace, Figure 2b) allows for a more faithful representation of the experimental fringe if compared to an overall data fitting using the theoretical shape for WGM resonances (red trace, Figure 2b), as shown in [19]. To take into account the resonance wobble induced by the flowing colloid, we adjusted the work-point of the parabolic approximation for each PA acquisition. Figure 3a shows the results of the transmission-detuning conversion for the zero-signal trace and the PA perturbed read-out trace of Figure 2c, keeping the same color code. In both cases the detuning associated with the work-point was subtracted (−297 MHz and −304 MHz, respectively) to emphasize the optical shift experienced by the WGM resonance due to the PA perturbation. About these optical shifts, we also underline that they are caused by the mechanical perturbation acting on the MBR walls, be it from the particle flow or the PA wave, rather than a perturbation of the refractive index of the colloidal solution contained in the MBR. In fact, the excited WGM is almost entirely confined within the MBR walls and therefore exhibits high sensitivity only towards effects that change the geometry or the refractive index thereof. The tiny evanescent tail in the core does not allow one to sense thermo-optical and/or piezo-optical effects induced in the colloidal solution by the PA wave.

Comparing the optical shifts shown in Figure 3a, a different trend can be noticed for the PA read-out traces, featuring an initial peak (at around 0.5 μs) followed by fast oscillations. These differences, which could be difficult to spot at first glance, are instead greatly highlighted when comparing the Fourier spectra of the traces (Figure 3b). The Fourier spectrum of the zero signal (black curve) is mostly flat with a little increase below 1 MHz, vaguely resembling an exponential decay; while the Fourier spectra of the PA read-outs (blue and red curves) are concentrated in a main peak at 5.75 MHz and in other minor peaks around 3.25, 4.75, and 8.5 MHz. Since the zero-signal spectrum (black curve) is substantially null above 1 MHz, the peaks associated with the PA read-out can be used as detection flags of PA emission, allowing the environmental noise to be effectively decoupled from the PA signal and to achieve a high signal-to-noise ratio (SNR). In particular, measuring PA wave intensity through the amplitude of the main Fourier peak at 5.75 MHz, instead of the peak-to-peak value of the time-domain trace, significantly increases the SNR, as shown by the 10 mJ/cm${}^{2}$ and the 5 mJ/cm${}^{2}$ fluence measurements of Figure 3a,b. In the first case, the SNR moves from 4.5 (1.8 vs. 0.4) for the time-domain trace to 18 ($67\cdot 10^{-3}$ vs. $3.7\cdot 10^{-3}$) for the 5.75 MHz Fourier peak, producing a four-time increase. In the second case, the time signal shows a less prominent peak and less intensely fast oscillations, making PA contributions more difficult to spot; however, the 5.75 MHz Fourier peak is still clearly visible with an SNR equal to 3.6. The ability to clearly distinguish between the PA signal and environmental noise as well as increasing the SNR in a challenging cytometry-like configuration are important results for the MBR system. In fact, these features are promising for a number of in vivo applications (e.g., measurement of blood cells oxygenation, detection of venous thrombi, and/or circulating tumor cells) where the noise source could be of physiological nature (e.g., heart beat, respiration), the excitation fluence could be limited to avoid tissue damage, and/or the amount of PA contrast agent could be limited to lower toxicity.

As discussed in [19] and in the associated Supplementary Materials, the peaks appearing in the Fourier spectrum are dictated by the mechanical spectrum of the MBR, since the WGM resonance shift is caused by the elastic deformation of the MBR walls induced by the PA wave. Figure 3c shows the deformation of the MBR walls (in arbitrary units) associated with a breathing mode close to 5.75 MHz. This modal shape was deduced by solving the eigenvalues problem through COMSOL Multiphysics^®^, taking into account the solid domain constituted by the MBR walls and the two fluid domains constituted by the GNR colloid and the air surrounding the MBR. The mechanical modes of the MBR are fundamental to achieving the decoupling of the signal from noise and the SNR improvement, but they also pose a limit to the application of the MBR transducer in PA imaging. Indeed, a PA imaging detector benefits from a flat frequency response in order to reconstruct the size of the PA emitters and different kinds of WGM detectors have been developed for this purpose [13,14,15].

**Figure 4:**
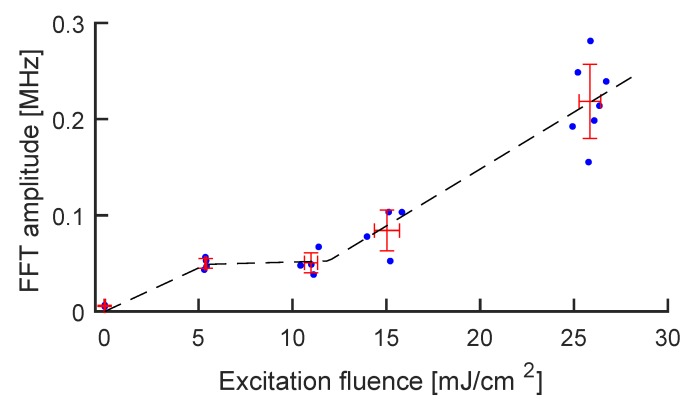
Amplitude of the main Fourier peak vs. pump laser fluence. The blue dots represent the experimental points, while the red crosses represent the average ± standard deviation for each group of measurements. The black curve is a guide to the eye showing the three lines associated with the three excitation regimes: regular PA generation, reshaping, and cavitation.

Finally, to demonstrate a quantitative reconstruction of the PA intensity through the height of the main peak at 5.75 MHz, we plotted this quantity against pump laser fluence in Figure 4. The typical photostability trend of GNRs can be recognized: a linear regime (up to about 5 mJ/cm${}^{2}$), when GNRs are stable under excitation and the PA response is linear with their absorbance; a sub-linear regime (5–10 mJ/cm${}^{2}$) associated with the onset of GNR reshaping [42,43]; a third regime (10–30 mJ/cm${}^{2}$), associated with the onset of cavitation regime, where shockwaves are produced through water evaporation in proximity of the GNRs [44]. Additionally, we challenged the MBR system by reducing the GNR concentration, obtaining an overall linear scaling of the photostability curve and therefore demonstrating sensitivity towards the concentration of the contrast agent (see Figure S2 of the Supplementary Materials).

## 4. Conclusions

We implemented a WGM microbubble resonator as an all-optical platform aimed at sensing the PA response of a flowing contrast agent, making a first step towards the implementation of this system for a cytometry application. As a result of the liquid flow through the MBR, the PA read-out was difficult to interpret on first glance and therefore we investigated its Fourier spectrum. We found that the PA signal could be easily decoupled from environmental noise since PA contributions were concentrated in sharp peaks. The amplitude of the most prominent Fourier peak was used as an indirect measurement of PA wave intensity and led to a significant increase in SNR. In terms of physical origin, a numerical solution of the mechanical eigenvalue problem for the MBR system allowed us to ascribe the Fourier peaks to the mechanical eigenmodes of the MBR. Finally, to demonstrate quantitative detection of PA intensity, we studied the amplitude of the main Fourier peak against pump laser fluence, finding the well-known photostability trend of GNR.

Noise decoupling and increased SNR are promising features for the MBR system with regard to a number of in vivo flow-cytometry applications (e.g., measurement of blood cells oxygenation, detection of venous thrombi, and/or circulating tumor cells), since they could reduce the effects of physiological noise (e.g., heart beat, respiration) and allow practitioners to work with lower excitation fluences and/or more diluted contrast agents. Additionally, the other MBR features, which are a low volume, direct implementation in a microfluidic circuit, and absence of acoustic impedance-matching material, are promising elements for wearable appliances and/or an endoscope.

Finally, on a more general note, all-optical PA detection allows for the interrogation of the material hosted in the MBR with a repetition rate that is ultimately limited by the mechanical decay time of the MBR eigenmodes. In our case, it would be possible to use an interrogation rate of up to 40 kHz (Figure 2c, blue curve), which for example, would allow the evolution of a chemical reaction with a 25 μs time step to be described by measuring the PA response of one of its reagents (e.g., growth of GNR, oxidation of the heme group).

## Supplementary Materials

The following are available online at https://www.mdpi.com/1424-8220/20/6/1696/s1.

## Abbreviations

The following abbreviations are used in this manuscript:

PA	Photoacoustic
WGM	Whispering Gallery mode
MBR	Microbubble resonator
GNR	Gold nanorods
