# Supplementary material for: Microbubble Resonators for All-Optical Photoacoustics of Flowing Contrast Agents
Source: Sensors (Basel). 2020 Mar 18;20(6):1696. doi: 10.3390/s20061696 (PMC7175143; doi:10.3390/s20061696)
Supplement: Supplementary file 1 [file sensors-20-01696-s001.pdf]

# Supplementary material: Microbubble resonators for all-optical photoacoustics of flowing contrast agents

Gabriele Frigenti [1,2,3], Lucia Cavigli [2], Alberto Fernández-Bienes [4], Fulvio Ratto [2], Sonia Centi [2], Tupak García-Fernández [5], Gualtiero Nunzi Conti [2,1] and Silvia Soria [2*]

1 Centro Fermi - Museo Storico della Fisica e Centro Studi e Ricerche "Enrico Fermi", Compendio del Viminale, Piazza del Viminale 1, 00184 Roma, Italy
2 CNR-IFAC, Istituto di Fisica Applicata "Nello Carrara", Consiglio Nazionale delle Ricerche, via Madonna del Piano 10, I50019 Sesto Fiorentino (FI), Italy
3 Laboratorio Europeo di Spettroscopia Nonlineare (LENS) - Università degli Studi di Firenze, via Nello Carrara 1, I50019 Sesto Fiorentino (FI), Italy
4 Facultad de Ingeniería, Universidad Nacional Autónoma de México (UNAM), Ciudad de México, C.P. 04510, México
5 Universidad Autónoma de la Ciudad de México (UACM), Prolongación San Isidro 151, Col. San Lorenzo Tezonco, México D.F., C.P. 09790, México
* Correspondence: s.soria@ifac.cnr.it

**Abstract**: This document expands the contents of the article *Microbubble resonators for all-optical photoacoustics of flowing contrast agents* by discussing auxiliary measurements.

---

### Supporting figures

Figure 1a shows the experimental set-up for the photoacoustic (PA) experiment with a standard piezoelectric transducer (Olympus Panametrics, mod V382-SU-F, sensor diameter 0.5 inch, frequency range 3.5 MHz, focal distance 0.83 inch, 40-dB amplifier mod 5676, Tokyo, Japan) as ultrasound detector and the same dispersion of gold nanorods (GNR), as reported in the main text, inside the microbubble resonator (MBR) as contrast agent. Both the transducer and the MBR are immersed in water (i.e. the acoustic-matching material). The MBR was kept in position using an holder with an aperture on the bottom, allowing the pump laser pulses provided by the Nd:Yag laser (Asclepion Laser Technologies, Jena, Germany; pulse duration 3.3 ns, repetition rate 10 Hz, pulse energy 40 µJ) to impinge on the MBR. The MBR itself is the little bulge in the yellow rectangle highlighting the resonator area. The recorded signal (blue trace in Figure 1b) features the typical dipolar shape associated with PA signals [1, 2, 3].

Figure 2 shows the height of the main Fourier peak (5.75 MHz) against the pump laser fluence for the GNR concentration used in the article (red data points, the same data appears in Figure 4 of the main text) and for its halved concentration (black data points). The measurements respect the concentration scaling within the experimental errors, except for the ones below 5 mJ/cm$^2$, which fall close to the limit-of-detection. This test shows that the MBR system is sensitive towards the concentration of the contrast agent, showing the validity of our experiment as a first step towards the implementation the MBR transducer into a real flow-cytometry application.

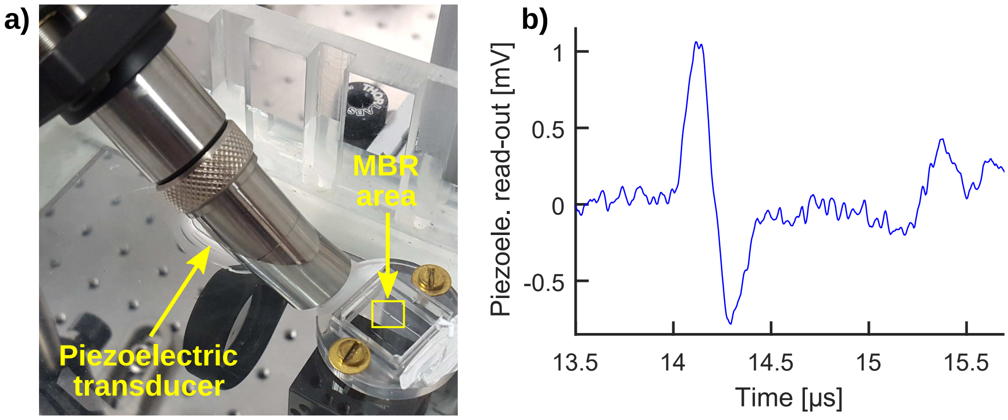


Figure 1: **a)** Photo of the experimental set-up: the transducer and the MBR (the little bulge in the zone delimited by the yellow rectangle) are both immersed in water (i.e the acoustic matching material). **b)** Signal recorded by the transducer, showing the typical features of PA signals.

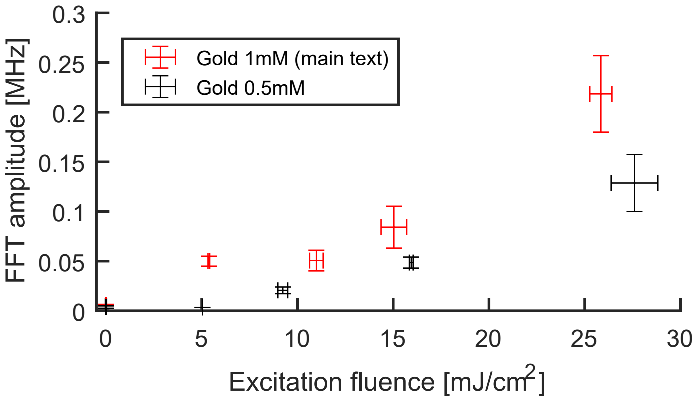


Figure 2: Height of the main Fourier peak at 5.75 MHz against pump laser fluence exciting the GNR. The trend is reported for two GNR concentration: the one used in the main text (red points) and its half.

**References**


[1] L. V. Wang, *Photoacoustic imaging and spectroscopy* (CRC press, 2009).

[2] P. M. Morse and K. U. Ingard, *Theoretical acoustics* (Princeton university press, 1986).

[3] A. Safari and E. K. Akdogan, *Piezoelectric and acoustic materials for transducer applications* (Springer Science & Business Media, 2008).
